# Characterization of the complete plastome of *Dendrophthoe pentandra* (Loranthaceae), a stem hemiparasite

**DOI:** 10.1080/23802359.2019.1667280

**Published:** 2019-09-19

**Authors:** Xiaorong Guo, Zhijie Ruan

**Affiliations:** a Institute of Ecology and Geobotany, Yunnan University, Kunming, Yunnan, China;; b School of Ecology and Environmental Science, Yunnan University, Kunming, Yunnan, China

**Keywords:** *Dendrophthoe pentandra*, Loranthaceae, hemiparasite, plastome

## Abstract

*Dendrophthoe pentandra* (Linnaeus) Miquel (Loranthaceae) is a stem hemiparasite distributed in southwest China and Indochina. Here, we present the first complete plastome of the plant. The plastome is 115,721 bp in length, and encodes 92 unique genes, including 63 protein-encoding gens, 4 rRNAs, and 25 tRNAs. Phylogenetic analysis indicated that *D. pentandra* is close to *Macrosolen cochinchensis* (Loureiro) Tieghem and *Helixanthera parasitica* Loureiro.

Because of the essential roles of chloroplast in green plants, their chloroplast genome (plastomes) are highly conserved in terms of genome size, structure, gene content, and organization (Daniell et al. 2016). However, the lifestyle transition from autotrophy to heterotrophy leads to varying degrees of size reduction, pseudogenization, gene loss, and structural rearrangement in the plastomes of parasitic plants (Wicke and Naumann 2018). Compared to holoparasitic plants, the plastomes of hemiparasites whose survive heavily relies on photosynthesis (Heide-Jørgensen 2008), have been poorly characterized. Therefore, the accumulation of plastome data will help explore the evolutionary pathway of plastome reduction associated with parasitism.

*Dendrophthoe* Martius, with 30 species from tropical Africa to Indochina and Australia, is a small genus in the family Loranthaceae A. L. Jussieu (Nickrent et al. 2010). To date, none plastome in this genus has been sequenced. In this study, we report the complete plastome of *Dendrophthoe pentandra* (Linnaeus) Miquel, a stem hemiparasites occurring in southwest China and Indochina (Qiu and Gilbert 2004).

Leaf tissues of *D. pentandra* were collected from Tengchong, Yunnan, China (N24°59′06.86″, E98°34′37.87″). Voucher (YLF-6) was identified by Dr. Yunheng Ji, and deposited in herbarium of Kunming Institute of Botany, Chinese Academy of Science (KUN). Genomic DNA was extracted from silica gel dried leaves. Purified DNA was sheared to fragments with an average length of 350 bp to construct shotgun library. The library was sequence on Illumina HiSeq 2500 system. We assembled the plastome using *Helixanthera parasitica* Loureiro plastome (MG808038) as reference, with CLC genome assembler v. 4.0β (CLC Inc., Aarhus, Denmark). The Dual Organellar Genome Annotator database (Wyman et al. 2004) was used to annotate plastome. Transfer RNA (tRNA) genes were identified by tRNAscan-SE 1.21 (Schattner et al. 2005) with the default parameters. The plastome sequence of *D. pentandra* was deposited in the NCBI GenBank database under the accession number MN175255.

The *D. pentandra* plastome is 115,721 bp in length, and presents a typical quadripartite structure with a large single-copy region (69,368 bp), a small single copy region (6,117 bp), and a pair of inverted repeat regions (20,118 bp). The plastome encodes 92 unique genes, including 63 protein-encoding gens, 4 ribosomal RNAs, and 25 tRNAs. Compared with the plastome of *Erythropalum scandens* Blume (NC_036759), a autotrophic plant of the sandalwood order, a total of 21 genes were deleted from the *D. pentandra* plastome, including 16 protein-coding genes (all the 11 *ndh* genes, *rps*15, *rps*16, *rpl*32, *rpl*36, and *inf*A), and 5 tNNAs (*trn*G-UCC, *trn*H-GUG, *trn*I-GAU, *trn*K-UUU, and *trn*V-UAC).

The relationships among Loranthaceae species were reconstructed based on complete plastome DNA sequences, using standard maximum likelihood (ML) method (Figure 1). *E. scandens* was selected as the outgroup. Plastome sequences were aligned using MAFFT (Kazutaka and Standley 2013). ML analysis was conducted using RAxML-HPC BlackBox v8.1.24 (Stamatakis 2006) with 1,000 replicates of rapid bootstrapping (BS). The phylogenetic analysis indicated that *D. pentandra* is closely related to *Macrosolen cochinchensis* (Loureiro) Tieghem and *Helixanthera parasitica*.

## Figures and Tables

**Figure 1.**
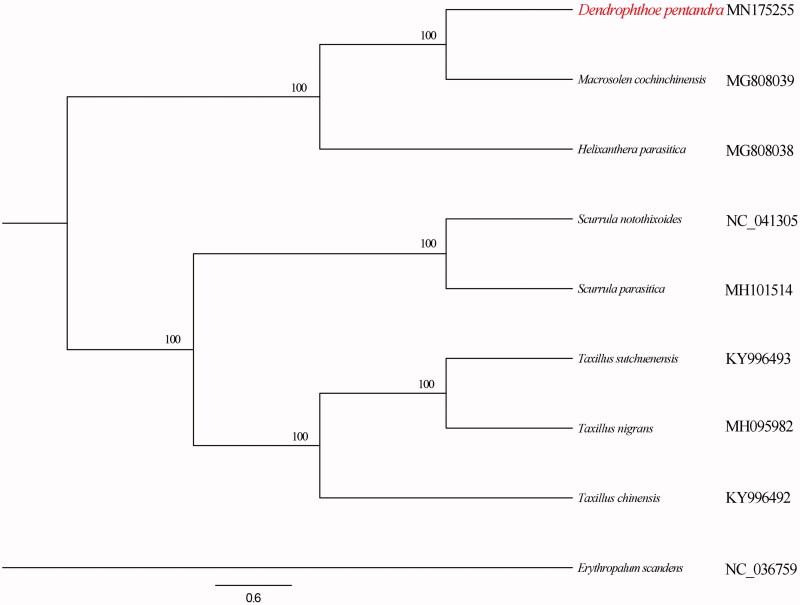
Phylogenetic relationships among Loranthaceae species, based on ML analysis of complete plastomes. The number on each node indicates bootstrap percentage.
